# 1-nonene plays an important role in the response of maize-aphid-ladybird tritrophic interactions to nitrogen

**DOI:** 10.3389/fpls.2023.1296915

**Published:** 2024-01-08

**Authors:** Shi-Wen Zhao, Yu Pan, Zhun Wang, Xiao Wang, Shang Wang, Jing-Hui Xi

**Affiliations:** ^1^ College of Plant Science, Jilin University, Changchun, China; ^2^ Plant Quarantine Laboratory, Changchun Customs Technology Center, Changchun, China

**Keywords:** nitrogen, tritrophic interactions, maize, 1-nonene, *Rhopalosiphum padi*, *Harmonia axyridis*

## Abstract

Plant volatile organic compounds (VOCs) are the key distress signals involved in tritrophic interactions, by which plants recruit predators to protect themselves from herbivores. However, the effect of nitrogen fertilization on VOCs that mediate tritrophic interactions remains largely unidentified. In this study, a maize (*Zea mays*)-aphid (*Rhopalosiphum padi)*-ladybird (*Harmonia axyridis)* tritrophic interaction model was constructed under high-nitrogen (HN) and low-nitrogen (LN) regimens. *H. axyridis* had a stronger tendency to be attracted by aphid-infested maize under HN conditions. Then, volatiles were collected and identified from maize leaves on which aphids had fed. All of the HN-induced volatiles (HNIVs) elicited an electroantennogram (EAG) response from *H. axyridis*. Of these HNIVs, 1-nonene was attractive to *H. axyridis* under simulated natural volatilization. Furthermore, our regression showed that the release of 1-nonene was positively correlated with *H. axyridis* visitation rates. Supplying 1-nonene to maize on which aphids had fed under LN enhanced attractiveness to *H. axyridis.* These results supported the conclusion that 1-nonene was the active compound that mediated the response to nitrogen in the tritrophic interaction. In addition, the 1-nonene synthesis pathway was hypothesized, and we found that the release of 1-nonene might be related to the presence of salicylic acid (SA) and abscisic acid (ABA). This research contributes to the development of novel environmentally friendly strategies to optimize nitrogen fertilizer application and to improve pest control in maize crops.

## 1 Introduction

Tritrophic interactions are among the most important components of all terrestrial ecosystems (Turlings and Erb, 2018). When under attack by herbivores, some plants recruit a third trophic level, which increases the plant’s attractiveness to the natural enemies of herbivores, and these organisms then provide the plant protection from herbivory and reduce plant damage (Hiltpold et al., 2011; Ali et al., 2023). Since evidence for an active role of herbivore-damaged plants in recruiting natural enemies of herbivores was first reported, an increasing number of different species of plants have been found to attract a range of herbivore enemies after an herbivore attack, including insect predators, parasitoids, predatory mites, nematodes, and birds (Clavijo et al., 2012; Meijer et al., 2023). Thus, tritrophic interactions are being increasingly discussed as an environmentally friendly crop protection strategy (Heil, 2008; Yang et al., 2023).

Volatile organic compounds (VOCs) serve as attractants for predators and parasitoids and play an important role in tritrophic interactions (Hiltpold et al., 2011; Aartsma et al., 2017; Turlings and Erb, 2018). Both green leaf volatiles (GLVs) and terpenoids attract predators or parasitoids of herbivores (Kessler and Baldwin, 2001). For example, (S)-(+)-linalool production was induced in *Oryza sativa* (rice) that had been attacked by *Spodoptera frugiperda* and in *Vicia faba* (broad bean) plants that were fed on by *Acyrthosiphon pisum* (pea aphids), and this compound attracted parasitic wasps. In addition, (L)-(+)-linalool produced by wild tobacco (*Nicotiana attenuata*) mediates a tritrophic interaction that involves *Manduca sexta* and predatory *Geocoris* spp. (big-eyed bugs) (He et al., 2019). In maize, GLVs are released in significant amounts after fresh damage to aboveground tissues (Turlings and Erb, 2018). The blend of volatiles emitted by maize plants attacked by noctuid species attracts parasitic wasps, such as *Cotesia marginiventris*, *Cotesia kariyai* (Turlings et al., 1990; Schnee et al., 2002; Kuramitsu et al., 2019). In underground tissues, β-b-caryophyllene is released in maize roots after damage by *Diabrotica virgifera* larvae to attract the entomopathogenic nematode *Heterorhabditis megidis* (Hiltpold et al., 2011). Thus, VOCs released by maize play important role in mediating interactions between pests and their enemies.

Nitrogen (N), which is one of the most important macronutrients for plants, plays a predominant role in the photosynthesis, growth, and development of plants; this has led to the application of N fertilizers as a key practice for increasing crop production (Chen et al., 2008; Kutyniok and Müller, 2013; Chesnais et al., 2016). In agroecosystems, the nutritional status of plants affects multitrophic plant-insect-predatory interactions (Jamieson et al., 2012). The response of tritrophic interactions between parasitoids and their host insects to nitrogen fertilizers has been researched extensively. Many studies have shown that improvement in N inputs enhances parasitism (Aqueel et al., 2014; Chesnais et al., 2016; Zhu et al., 2020a) and influences the growth and development of both pests and their parasites (Kaneshiro et al., 1996; Aqueel et al., 2014). In addition, nitrogen levels affect the levels of plant hormones and the expression of numerous genes involved in metabolite synthesis, which subsequently results in changes in plant volatiles that might be the key signals used by natural enemies to find pests (Chen et al., 2008; Kutyniok and Müller, 2013). For example, rice plants treated with different nitrogen levels showed changes in the composition of volatiles, which regulated the foraging and searching behavior of *Cyrtorhinus lividipennis* (Lou and Cheng, 2003; Zhu et al., 2020b). Although it has been speculated that a higher N level would increase the attractiveness of plants to natural enemies via plant-based cues (Loader and Damman, 1991; Zhu et al., 2020a), the relative importance of individual compounds in mediating the response of tritrophic interactions to nitrogen is still poorly characterized.

As the most widely grown crop, maize is an excellent subject for studying the effects of nitrogen fertilization on tritrophic interactions (Jagtap et al., 2020). The aphid *Rhopalosiphum padi* (L.) is a major herbivore of maize in many countries (Chirgwin et al., 2022). *Harmonia axyridis* Pallas (Coleoptera: Coccinellidae) is widely reported as a natural enemy of *R. padi* (Kontodimas and Stathas, 2005). Therefore, it is necessary to study the interactions among these three species. In this study, we explored how nitrogen fertilization impacted the ability of maize plants infected with *R. padi* to attract *H. axyridis*. Furthermore, the active compounds mediating the response of the maize-*R. padi*-*H. axyridis* tritrophic interactions with nitrogen were verified. These results might help in the development of novel strategies to optimize nitrogen application, improve yields, and facilitate biological control of pests of the important crop maize.

## 2 Materials and methods

### 2.1 Plant materials

The maize cultivar ‘B73’ was sown in plastic pots (height, 20 cm; diameter, 15 cm) that contained a mixture of sterilized field soil and sand (3:1) (Schnable et al., 2009). The soil was nutrient-poor (organic matter 31.67 g/kg, total nitrogen 0.716 g/kg, total phosphorus 0.135 g/kg, and available nitrogen 5.86 mg/kg). Fertilization was started on day seven of the experiment with a modified Hoagland solution. Depending on the N treatment scheme, the nutrient solution contained 15 mM KNO_3_ (for the HN treatment) or 0.15 mM KNO_3_ (for the LN treatment) (Schluter et al., 2012). The seedlings were grown in a climate chamber (22 ± 2°C, 70% relative humidity, 16 h:8 h light:dark). Experimentation began 20 d (three leaf stage) after germination.

### 2.2 Insect rearing

A population of *R. padi* was obtained from a single individual. *R. padi* and *H. axyridis* were reared in a Perspex rearing cabinet under controlled conditions (22 ± 2°C, 70% relative humidity, 16 h:8 h light:dark). *R. padi* and female *H. axyridis* were obtained from the Agricultural Experiment Base of Jilin University (Changchun, Jilin, China) (Qin et al., 2021).

### 2.3 Collection and identification of maize VOCs

VOCs from leaves of aphid-infested maize under high- and low-nitrogen conditions were collected using a dynamic headspace solid-phase microextraction (SPME) method (Pan et al., 2021). Maize leaves without aphid honeydew were strictly screened out for the experiment to avoid interference from aphid honeydew. Twenty adult aphids were allowed to feed on maize plants for 24 h and later removed before collection. A 0.5 g sample of leaves from each of those two groups was placed into a 50 mL glass bottle. An SPME fibre that was coated with polydimethylsiloxane-divinylbenzene (PDMS-DVB, 65 mm), purchased from Supelco (Bellefonte, PA, U.S.), was conditioned at 250°C for 30 min in a gas chromatograph injection port, according to the manufacturer’s guidelines. Plants were kept in a glass container for 1 h before sampling, headspace sample from the empty glass container was collected as controls, and the controls were used to eliminate impure volatiles emitted by the headspace collecting instrument. The SPME fibre was inserted into the opening of the glass container, and the fibre was extended to absorb the plant volatiles. After 1 h, the SPME fibre was inserted directly into a gas chromatograph-mass spectrometer (GCMS-QP2010Ultra, SHIMADZU, Japan) equipped with an Rxi‐5MS capillary column (30 m length, 0.32 mm i.d., 0.25 µm film thickness). The fibre was inserted into the injector port at 250°C and desorbed for 5 min. After fibre insertion, the column temperature was maintained at 40°C for 1 min and then increased to 250°C at 10°C min ^-1^, followed by a final stage of 4 min at 250°C. Six replicates were included for each treatment. The volatile compounds were identified by matching their retention times to those of authentic standards, as well as by comparing the MS spectrum fragmentation patterns to the NIST08 Mass Spectral Library (National Institute of Standards and Technology, Washington D.C., U.S.) and authentic standards (Ye et al., 2018; Pan et al., 2021). The Kovats retention index of each volatile component was calculated using the retention time, and the data were matched to previously published data. The peak area of each component of the volatiles, which were tentatively identified, represented the relative quantity (Hiltpold et al., 2011).

The authentic standards benzaldehyde (98%), acetic acid (98%), 1-nonene (95%), and indole (98%) were purchased from Sigma Aldrich (Deisenhofen, Germany) and dissolved in hexane for the subsequent assay. The amount of volatiles emitted in the sample headspace was determined by a semiquantitative method (Erb et al., 2015). One-microlitre samples of the authentic compound standards at five concentrations (10 ng/μL, 25 ng/μL, 50 ng/μL, 100 ng/μL, and 200 ng/μL) were injected into the liquid chromatograph. The standard curve and linear regression equation were calculated by quantifying 1 μL samples of authentic compound standards at various concentrations and measuring the corresponding peak areas from liquid chromatography. The amounts of the of natural volatiles emitted in the headspace sample were calculated using the linear regression equation with the corresponding peak areas.

### 2.4 *H. axyridis* Y-maize assays

A Y-tube olfactometer was used to determine the behavioral responses of *H. axyridis* to aphid-infested maize under different nitrogen levels. Prior to each experiment, all glassware was washed with distilled water and baked in an oven overnight at 160°C. A 20 W fluorescent light was placed 0.5 m above the olfactometer. Host-related behaviour was examined as follows: to examine the behavioral response of *H. axyridis* to maize fed on by aphids under high/low-nitrogen conditions, two choice tests were performed using a glass Y-tube olfactometer (20 cm×20 cm×20 cm arm length, 4 cm diameter, 75°Y angle). The tests included 1) healthy maize under HN vs. healthy maize under LN, 2) aphid-infested maize under LN vs. healthy maize under LN, 3) aphid-infested maize under HN vs. healthy maize under LN, and 4) aphid-infested maize under HN vs. aphid-infested maize under LN. Plants were kept in a glass container for 1 h before bioassays to eliminate any contaminating volatiles from the system. Aphids were removed prior to the experiment to eliminate the effects of their volatiles. *H. axyridis* was introduced into the main arm of the Y-tube olfactometer. Air was then blown through two glass containers and then into the two side arms at 400 mL/min. *H. axyridis* was considered to have made the first choice when it moved > 3 cm into either arm (visually assessed by a line marked on both arms). The final choice for *H. axyridis* was the arm it was in at the end of the 5-min experimental period. We excluded *H. axyridis* individuals that did not make any choice within 5 min from the data analysis. *H. axyridis* females were placed individually in the Y-tube for the behavioral assay. The experiment had six replicates in total, ten females and new plants were used per replicate For each replicate, 5 females were moved in one direction and the others were moved in the other direction to control for any directional bias in the room. Each insect was used only once (Gouinguené et al., 2005).

The responses of *H. axyridis* to a single compound were also determined using a Y-tube olfactometer as described above. The compounds in the dispensers placed in the treated arm were allowed to evaporate for 1 h before insect placement; the control dispenser was placed in the control arm. As previously described (Pan et al., 2021), the dispensers consisted of 2-mL amber glass vials containing 1 mL of authentic compounds (106.5 ng/μL 1-nonene, 149.9 ng/μL acetic acid, 15.04 ng/μL indole, and 15.13 ng/μL benzaldehyde). The vials were sealed with open screw caps that contained a rubber septum, which was pierced with a 2-μL micropipette tip. Control dispensers were prepared in the same way with 1 mL of hexane. The amounts of compounds that the dispensers released approximately corresponded to those of high nitrogen-induced volatiles (HNIVs) emitted by maize.

### 2.5 Electroantennogram response of *H. axyridis*

An electroantennogram (EAG) assay was performed to measure the sensitivity of *H. axyridis* to benzaldehyde, 1-nonene, acetic acid, azulene, indole, caryophyllene, and (-)-aristolene. Solutions of single synthetic compounds were diluted in distilled paraffin oil. The antennae of *H. axyridis* insects were removed carefully at the base, and several terminal segments at the distal end were excised before attaching them to electrodes using Spectra 360 conductive gel (Parker, Fairfield, USA). Test compounds (i.e., 10 μL, 10 μg/μL) were applied to a piece of filter paper, which was inserted into a syringe. The strip was placed in the syringe, which delivered a continuous humidified (60–70%) air flow (500 mL/min) and added a compensatory flow. The duration of the stimulation was 0.1 s, and the signal from each antenna was recorded for 4s. Two minutes intervals were allowed between stimulations to restore EAG sensitivity. At least five individuals were tested, and each individual was tested three times. The response to the reference standard (*z*)-3-hexen-1-ol was measured at the beginning and end of each recording session to correct for the loss of sensitivity in the preparation. For correction, it was assumed that the decrease in sensitivity was linear over time. The data were then normalized to the standard as follows (Jing et al., 2021):


$$\text{rEAG}=\frac{\text{EAG}\left(\text{A}\right)}{\text{EAG}\left(\text{std}1\right)+\frac{\text{EAG}\left(\text{std}2\right)-\text{EAG}\left(\text{std}1\right)}{\text{RT}\left(\text{std}2\right)-\text{RT}\left(\text{std}1\right)}\times \left(\text{RT}\left(\text{A}\right)-\text{RT}\left(\text{std}1\right)\right)}$$,

where rEAG is the relative EAG response, EAG(A) is the amplitude (mV) of the EAG response to compound A, EAG(std1) is the EAG response to the standard at the beginning of the recording, EAG(std2) is the EAG response to the standard at the end of the recording, T(A) is the time elapsed before stimulation with compound A, T(std1) is the time of the first stimulation, and T(std2) is the time of the final stimulation.

### 2.6 *H. axyridis* tent assays

To test the visit number of *H. axyridis*, tent (2 m × 2 m × 1.5 m) assays were conducted. The four treatments in the four corners of the tents were healthy maize under HN, healthy maize under LN, aphid-infested maize under HN, and aphid-infested maize under LN. There were six replicates, and 30 females were used for each replicate. Each replicate was conducted for 15 min. The number of *H. axyridis* individuals that visited each of the four treatments was recorded. The location of four treatments was randomly arranged for each replicate (Zhang et al., 2022).

### 2.7 Verification of active compounds using repeated bioassays

The tent assays were conducted again to determine the behavioral responses of *H. axyridis* to 1-nonene and acetic acid in the repeated bioassays as described above. The four treatments, including aphid-infested maize under LN, aphid-infested maize under HN, aphid-infested maize under LN supplied with 1-nonene, and aphid-infested maize under LN supplied with acetic acid, were placed at the four corners.

A Perspex four-arm olfactometer was used to determine the behavioral responses of *H. axyridis* to 1-nonene in the repeated bioassays. Four treatments with 0.5 g of leaves or compound dispensers (used to supply 1-nonene and acetic acid) were placed in 50 mL bottles that were connected directly to the four olfactometer chamber arms. The compound dispensers were the same as those described for the Y-tube. The four treatments included aphid-infested maize under LN, aphid-infested maize under HN, aphid-infested maize under LN supplied with 1-nonene, and aphid-infested maize under LN supplied with acetic acid (control) at each of the four sides of the olfactometer. An airstream at 400 mL per min was created by removing air from the center of the chamber with a vacuum pump. A 20 W fluorescent light was placed 0.5 m above the olfactometer. To avoid visual distraction of *H. axyridis*, a white curtain was placed around the olfactometer. Air was passed through a charcoal filter to remove any impurities. Filtered air flowed over the odor source into each of the four arms towards the center of the chamber. A single *H. axyridis* individual was introduced into the center of the chamber and observed for 5 min. If it walked up in the direction of the olfactometer and into one arm, it was recorded and removed. If an *H. axyridis* did not choose an arm within 5 min, it was considered nonresponsive and discarded. The experiment had six replicates, and 30 *H. axyridis* were used for each replicate. The olfactometer was rotated 90° after 5 *H. axyridis* individuals were tested. All equipment was cleaned before use (Ye et al., 2018; Zhang et al., 2022).

### 2.8 Quantitative RT−PCR

All RNA was extracted from maize-leaves using RNAiso Plus (Takara, Dalian, China) according to the manufacturer’s protocol. The PrimeScript™ RT Reagent Kit with gDNA Eraser (RR047A, Takara, Dalian, China) was used for cDNA synthesis. Specific primer pairs for RT−qPCR for selected key genes in the synthesis pathways from the maizeGDP website were designed using Primer Premier 5.0 software (**Supplementary Table 1**). *Actin* (GenBank accession number: J01238) was used as a candidate reference gene (Manoli et al., 2012). The PCR conditions were as follows: 95°C for 30 s, followed by 40 cycles of 94°C for 5 s, 60°C for 10 s, and 72°C for 34 s. The reaction mixture (final volume of 20 μL) contained 1 μL of cDNA, 10 μL of SYBR Premix Ex Taq, 0.4 μL of 10 μM forward primer, 0.4 μL 10 μM of reverse primer, 0.4 μL of ROX Reference Dye II, and 7.8 μL of double-distilled water. After RT−qPCR, melting curves were evaluated to confirm single peaks and to check amplification specificity. Subsequently, the relative expression level was calculated using the 2^−ΔΔCt^ method (Pfaffl, 2001). The reaction was performed with three biological replicates and three technical replicates (Pan et al., 2021).

### 2.9 Phytohormone analysis

Maize leaves samples (approximately 100 mg) were harvested and transferred to FastPrep tubes. One milliliter of ethyl acetate spiked with 200 ng of d5-JA, d6-ABA, and d4-SA was added to each sample (n = 9) and used as the internal standards for JA, ABA, and SA, respectively. Ten-microlitre aliquots of the samples were analyzed using a triple quadrupole liquid chromatography with tandem mass spectrometry (LC−MS/MS) system (Shimadzu LC-20A coupled with an Applied Biosystems API4000 mass spectrometer); this apparatus was equipped with an SB-C18 column (2.1 mm×150 mm, 3.5 µm; Agilent Technologies) and kept in a thermostat-controlled chamber at 35°C. A mobile phase composed of solvent A (0.1% formic acid) and solvent B (acetonitrile) was used in gradient mode for separation at a constant flow rate of 0.2 mL min^−1^. The compounds were detected in electrospray ionization negative mode. JA, SA, and ABA were quantified by comparing their peak areas with the peak areas of their respective internal standards (Pan et al., 2021).

### 2.10 Statistical analysis

Statistically significant differences for the volatile were using Student’s *t*-test. Y-tube olfactometer bioassays were analyzed with a Shapiro-Wilk test to determine heteroscedasticity of error variance and normality, significance of the linear regressions (*p*< 0.10), the data conform to normal distribution, so Y-tube olfactometer bioassays were analyzed using a paired *t*-test, Multiple comparisons of phytohormone data were performed using Tukey’s test. Multiple comparisons of EAG data were performed using Dunnett’s test. The averages of the amounts of emitted volatiles and phytohormone from the different treatment groups were used for correlation analysis. The released volatiles and the number of *H. axyridis* visits was conducted using a simple regression. The amounts of VOCs emitted from aphid-infested maize in HN and LN are listed in **Table 1**, and the amounts of VOCs emitted from healthy maize in HN and LN were obtained from a previous article from our laboratory (Zhao et al., 2022). IBM SPSS statistics version 20 (Chicago, IL, U.S.) was used to conduct above statistical analyses. Principal component analysis (PCA) and random forest analysis were conducted by the online program MetaboAnalyst (https://www.metaboanalyst.ca/). Generalized linear mixed models (GLMM) with Poisson distribution were performed to test difference in the four-arms bioassays and the tent bioassays using R version 4.2.1 (R Core Team, 2022). The GLMM were analyzed with lme-function of lme4 package. For *post hoc* analysis of the above data, the multiple comparisons were performed and the *p*-value were calculated using Holm’s tests. The random effect analysis for the replication was conducted, there is no random effects of replication in the assays.

**Table 1:** Volatile organic compounds (VOCs) emitted from aphid-infested-maize leaves under high and low nitrogen conditions.

Compound	High nitrogen condition	Low nitrogen condition	
Farnesol	0.23 ± 0.07	0.16 ± 0.06	NS
1-Nonene	0.37 ± 0.08	0.13 ± 0.01	***
Benzaldehyde	0.79 ± 0.09	0.54 ± 0.10	*
Squalane	0.30 ± 0.09	0.19 ± 0.05	NS
Longifolene	0.22 ± 0.14	0.16 ± 0.04	NS
Eicosane	0.17 ± 0.04	0.18 ± 0.07	NS
Ylangene	0.77 ± 0.14	0.78 ± 0.27	NS
Naphthalene	0.62 ± 0.11	0.90 ± 0.18	NS
Copaene	2.13 ± 0.38	2.08 ± 0.36	NS
Caryophyllene	0.01 ± 0.01	0.57 ± 0.19	***
Azulene	0.44 ± 0.05	0.75 ± 0.08	*
alpha-Cubebene	0.30 ± 0.19	0.28 ± 0.18	NS
(+)-Cycloisosativene	0.25 ± 0.12	0.21 ± 0.11	NS
(-)-Aristolene	0.16 ± 0.1	0.41 ± 0.19	*
Indole	0.49 ± 0.09	0.14 ± 0.04	***
Acetic acid	0.24 ± 0.09	0.01 ± 0.01	***
Hexadecanoic acid	0.28 ± 0.03	0.27 ± 0.04	NS

The relative amounts of volatile compounds (mean Area% ± s.e, n = 6). NS indicates no significant difference. Asterisks indicate statistically significant differences under high nitrogen conditions compared with low nitrogen conditions. The nutrient solution contained 15 mM for the HN treatment or 0.15 mM KNO3 for the LN treatment. (Student’s t-test: * p< 0.05; ** p< 0.01; *** p< 0.001).

## 3 Results

### 3.1 High nitrogen levels enhanced the ability of infected maize to attract *H. axyridis*

**Figure 1:**
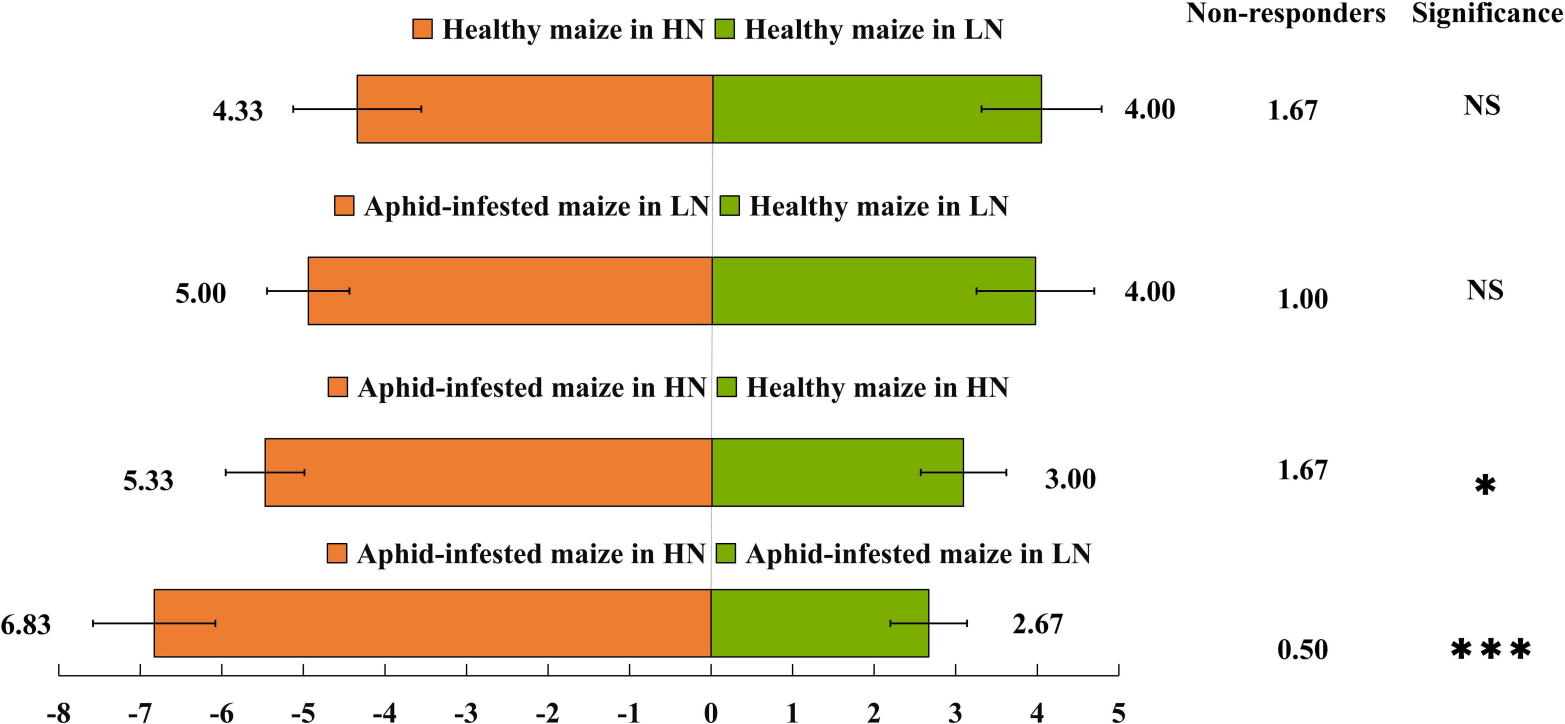
Behavioural responses of Harmonia axyridis. A Y-tube olfactometer was used to determine the behavioral responses of H. axyridis to maize under different treatments: healthy maize under high-nitrogen (HN) conditions vs. healthy maize under low-nitrogen (LN) conditions; healthy maize under low-nitrogen conditions vs. aphid-infested maize under low-nitrogen conditions; healthy maize under high-nitrogen conditions vs. aphid-infested maize under high-nitrogen conditions; and aphid-infested maize under high-nitrogen conditions vs. aphid-infested maize under low-nitrogen conditions. There are six replicates in the Y-tube olfactometer assays, 10 H. axyridis individuals were used for each replicate. (t-test: *p< 0.05; ***p< 0.001, NS, not significant).

The Y-tube olfactory behavior experiment showed that under HN conditions, *H. axyridis* preferred aphid-infested maize to healthy maize (*t* = 4.464; *df* = 10; *p* = 0.001), furthermore, *H. axyridis* was attracted by aphid-infested maize under HN conditions rather than LN conditions (*t* = 9.19; df = 10; *p*< 0.0001) (**Figure 1**). However, nitrogen level did not affect their trend to healthy maize (*t* = 0.439; *df* = 10; *p* = 0.67), besides, aphid infestation did not affect their trend to maize under LN conditions (*t* = 0.341; *df* = 10; *p* = 0.145). That is, we concluded that *H. axyridis* used the VOCs of maize to locate this crop, moreover, aphids infestation had effect on the *H. axyridis* choice to maize under HN conditions and nitrogen level affect the attraction of aphid-infested maize to the *H. axyridis.*

### 3.2 Identification of differential VOCs from aphid-infested maize under high- and low-nitrogen conditions

**Figure 2:**
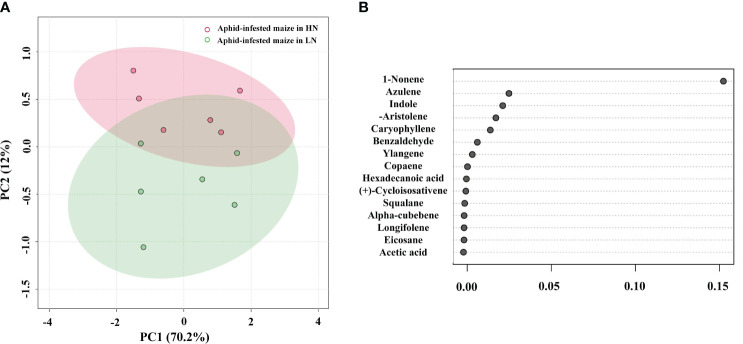
**(A)** Principal component analysis of the volatile organic compounds (VOCs) of the aphid-infested maize under high-nitrogen (HN) and low-nitrogen (LN) conditions. **(B)** Features of VOCs ranked by their contributions to classification accuracy (mean decrease accuracy).

To identify the volatiles emitted by the maize plants that triggered indirect defense, samples of the volatiles were collected from infested plants. GC−MS analysis detected 17 major VOCs from maize under high/low-nitrogen conditions (**Table 1**). Seven of these volatile compounds showed significant differences between HN and LN conditions. Benzaldehyde (*t* = 4.464; *df* = 10; *p*< 0.05), 1-nonene (*t* = 41.003; *df* = 10; *p*< 0.01), acetic acid (*t* = 5.921; *df* = 10; *p*< 0.001), and indole (*t* = 8.673; *df* = 10; *p*< 0.001) were more abundant under HN than under LN. (-)-Aristolene (*t* = 2.9; *df* = 10; *p*< 0.05), azulene (*t* =8.161; *df* = 10; *p*< 0.0001), and caryophyllene (*t* = 7.281; *df* = 10; *p*< 0.0001) were produced at higher amounts under LN than under HN. The relative amounts of other VOCs were not significantly different (**Table 1**). PCA of the VOCs showed significant separation between maize infested by aphids in HN and LN conditions. PC1 and PC2 accounted for 82.2% of the variation (**Figure 2A**). Random forest distribution function analysis showed that 1-nonene had the highest absolute scores (**Figure 2B**), suggesting that 1-nonene made high contributions to the classification accuracy.

### 3.3 Induced VOCs from aphid-infested maize under high-nitrogen conditions elicited the EAG responses of *H. axyridis*

**Figure 3:**
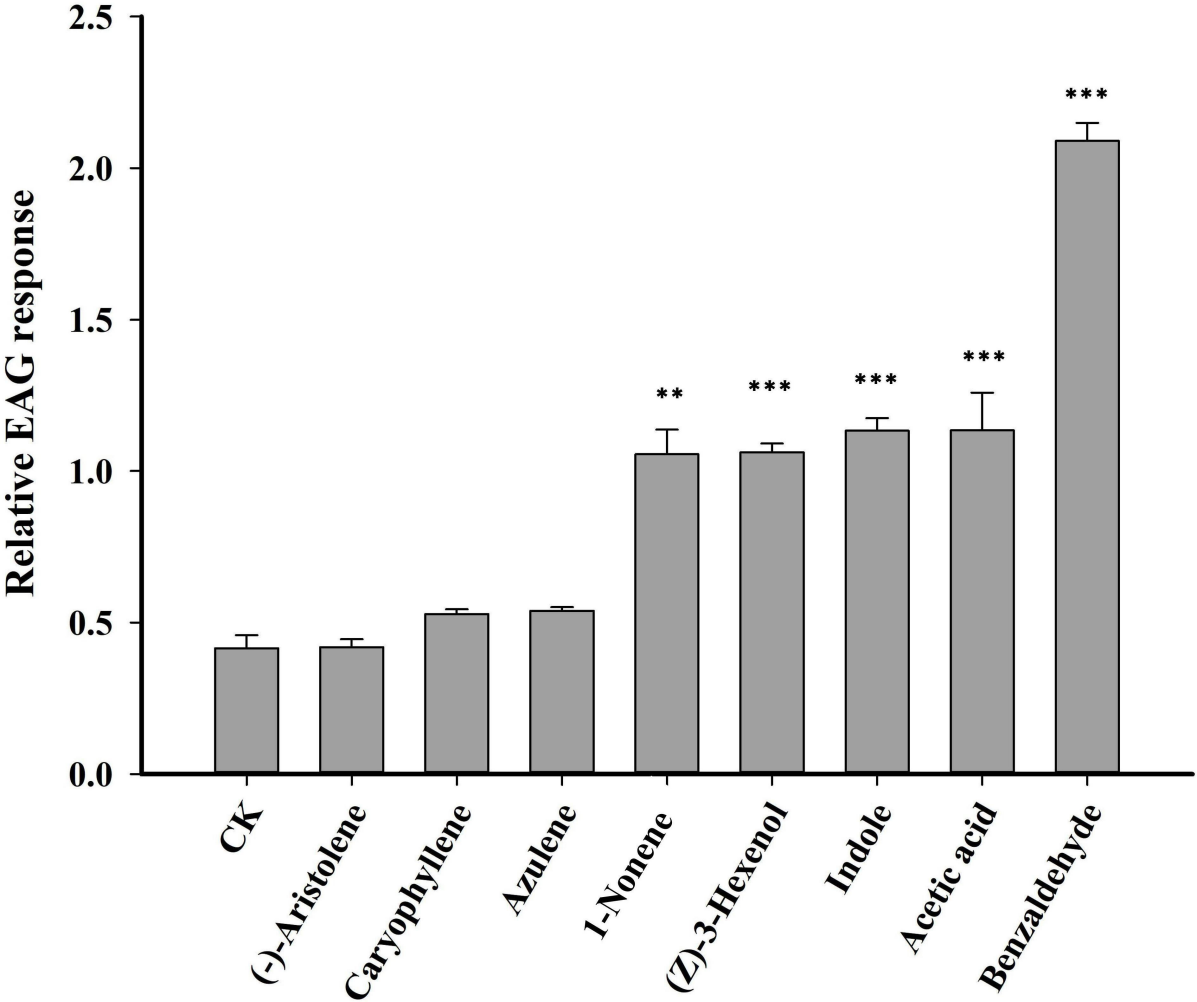
An electroantennogram assay was performed to measure the sensitivity of H. axyridis to CK (paraffin oil), benzaldehyde, 1-nonene, acetic acid, azulene, indole, and (-)-aristolene. The EAG multiple comparisons was used Dunnett’s test (**p< 0.01; ***p< 0.001).

Seven VOCs that significant differences between HN and LN conditions were selected for EAG experiments. Of the seven VOCs, four VOCs (benzaldehyde, 1-nonene, indole and acetic acid) elicited the significantly greater EAG responses of *H. axyridis*, the EAG response values of benzaldehyde (*p*< 0.0001), 1-nonene (*p* = 0.008), indole (*p*< 0.0001), and acetic acid (*p*< 0.0001) were significantly higher than the control (paraffin oil) (**Figure 3**), however, the *H. axyridis* didn’t show the significantly greater EAG responses to (-)-aristolene (*p* = 1), caryophyllene (*p* = 0.531), and azulene (*p* = 0.697) compared to control (**Figure 3**). The four HIPVs that were more strongly emitted under high N conditions may have affected *H. axyridis* response.

### 3.4 Behavioural responses of *H. axyridis* to differential VOCs

**Figure 4:**
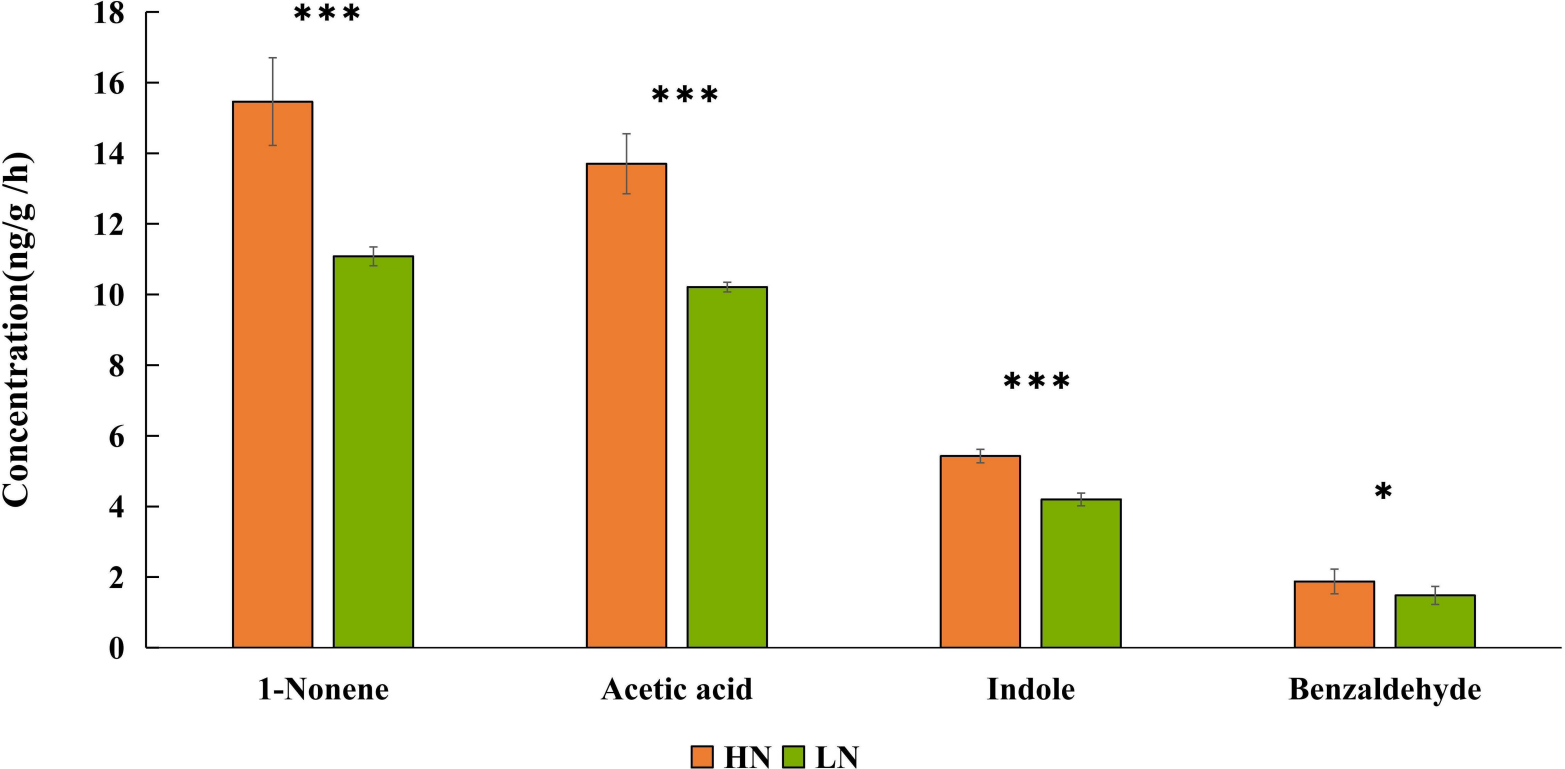
Quantitative analysis of 1-nonene, acetic acid, indole, benzaldehyde emitted by aphid-infested maize under high-nitrogen conditions/low-nitrogen conditions. Individual compounds with authentic standards at known concentrations were used for the determination of the absolute amount of each active compound in the headspace volatile sample. The data are shown as the mean ± standard error (SE). Asterisks indicate statistically significant differences between HN and LN (Student’s t-test: *p< 0.05 ***p< 0.001).

Based on the EAG results, four compounds (1-nonene, acetic acid, indole, and benzaldehyde) were selected for behavioral assay. The emissions of these four compounds were quantified from aphid-infested maize under HN condition (**Figure 4**), and the volatilization rates are as follows: 15.46 ng/g/h 1-nonene, 13.70 ng/g/h acetic acid, 5.43 ng/g/h indole, and 1.88 ng/g/h benzaldehyde. The volatilization rates of 1-nonene (*t* = 20.276; *df* = 4; *p*< 0.0001), acetic acid (*t* = 17.08; *df* = 4; *p*< 0.0001), indole (*t* = 92.5; *df* = 4; *p*< 0.0001), and benzaldehyde (*t* = 3.331; *df* = 4; *p* = 0.0291) were significantly higher than those from aphid-infested maize under LN conditions. Then, we investigated the effects of synthetic standards, which were used at the natural concentrations emitted by HN aphid infested maize, on the behavior of *H. axyridis* using a Y-tube olfactometer. The results indicate that 1-nonene exhibited a significantly attractive effect on *H. axyridis* compared to the control (*t* = 3.337; *df* = 10; *p* = 0.008), however, benzaldehyde (*t* = 1.936; *df* = 10; *p* = 0.082), indole (*t* = 2.076; *df* = 10; *p* = 0.065) and acetic acid (*t* = 0.275; *df* = 10; *p* = 0.789) was not exhibited attraction to *H. axyridis* (**Figure 6**).

### 3.5 Regression analysis between VOCs and visits of *H. axyridis*

To further validate that high nitrogen levels enhanced the ability of infected maize to attract *H. axyridis*, the tent bioassays were conducted, the number of *H. axyridis* visiting aphid-infested maize under HN conditions was significant higher than those of *H. axyridis* visiting healthy maize under HN conditions (*p* = 0.006), aphid-infested maize under the LN conditions (*p* = 0.006), as well as healthy maize under the LN conditions (*p* = 0.006). However, there were no significant differences between healthy maize and aphid-infested maize under LN conditions (*p* = 0.579), between the healthy maize under LN conditions and healthy maize under the HN conditions (*p* = 1), and between healthy maize under HN conditions and aphid-infested maize under LN (*p* = 1) (**Figures 6A, B**), which is consistent with the results of Y tube. In order to evaluate the responses of *H. axyridis* to the four compounds, which were identified as potential active compounds and known to be influenced by nitrogen levels, we performed a regression analysis between the peak area of each of the four compounds and the number of *H. axyridis* visits (**Figure 6C**). The number of *H. axyridis* visits increased along with the increase of peak area of 1-nonene (R^2 ^= 0.954, *F* = 41.588, *p* = 0.023) (**Figure 6C**). But the number of *H. axyridis* visits were not significantly changed with increase of the peak area of indole(R^2 ^= 0.174, *F* = 0.423, *p* = 0.582), acetic acid (R^2^< 0.0001, *F*< 0.0001, *p* = 0.998) and benzaldehyde(R^2 ^= 0.106, *F* = 0.237, *p* = 0.675) (**Figure 6C**).

### 3.6 Verification of the ecological potential of 1-nonene using repeated bioassays

**Figure 5:**
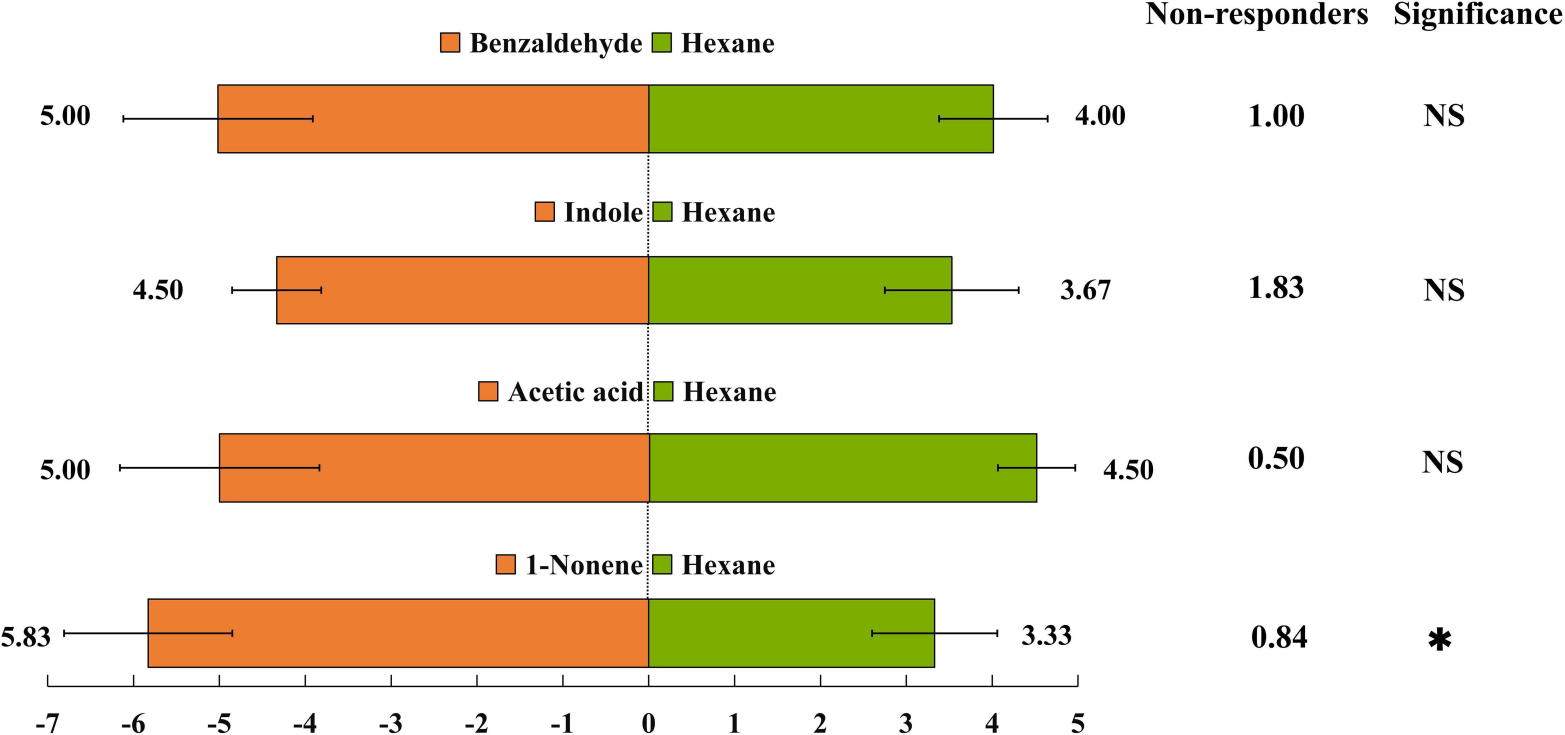
Behavioural responses of Harmonia axyridis to synthetic chemicals were determined using a Y-tube olfactometer. Synthetic chemicals, including 1-nonene, acetic acid, indole, and benzaldehyde, correspond to amounts typically emitted by aphid-attacked maize under high-nitrogen conditions. There are six replicates in the Y-tube olfactometer assays, 10 H. axyridis individuals were used for each replicate. (t-test: *p< 0.05, NS, not significant).

Four-arm olfactometers and tent assays were used in repeated bioassays to evaluate the ecological potential of 1-nonene. The volatilization of 1-nonene and acetic acid from aphid-infested maize under HN was 4.38 ng/g/h and 3.49 ng/g/h higher, respectively, than that from aphid-infested maize under LN, which are derived from the results in **Figure 5**. Aphid-infested maize under LN supplemented with the missing portion of 1-nonene and acetic acid was used in the tent assays and four-arm olfactometers. The aphid-infested maize under LN supplemented with 1-nonene significantly attracted more *H. axyridis* than the aphid-infested maize under LN in tent assays (*p* = 0.006) and in four-arm olfactometers (*p* = 0.012), and showed similar attractiveness as the aphid-infested maize under HN in tent assays (*p* = 0.414) and in four-arm olfactometers (*p* = 1). However, compared to the aphid-infested maize under LN, *H. axyridis* was not attracted by the aphid-infested maize under LN supplemented with acetic acid in tent assays (*p* = 0.414) and in four-arm olfactometers (*p* = 0.1) (**Figure 7**).

**Figure 6:**
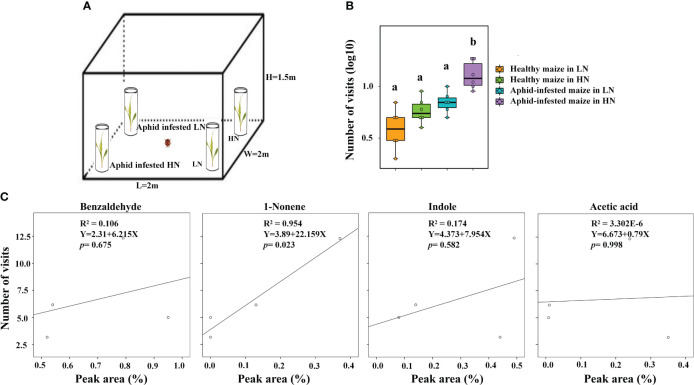
The analysis between VOCs and visits of H. axyridis. **(A)** Schematic drawing of the tent assay. **(B)** Number of visits (log10) of H. axyridis in four different treatments, including healthy maize in high nitrogen (HN), healthy maize in low nitrogen (LN), aphid-infested maize in HN, and aphidinfested maize in LN. Different letters above the bars indicate significant differences (The analyzed using a GLMM fitted to a poisson distribution). **(C)** The effect of the amount (Peak area %) of volatiles on attraction (visit number) evaluated using a simple regression analysis.

**Figure 7:**
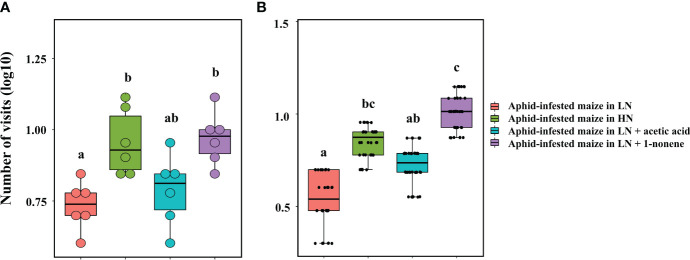
Behavioural responses of *H*. *axyridis* to aphid-infested maize supplied with 1-nonene under low nitrogen conditions in the tent bioassay **(A)** and four-arm olfactometer **(B)**. LN, low nitrogen; HN, high nitrogen. Different letters above the bars indicate significant differences (The analyzed using a GLMM fitted to a poisson distribution).

### 3.7 The regulation pathway of 1-nonene

**Figure 8:**
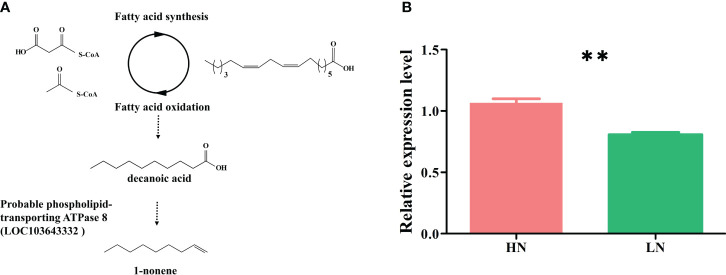
**(A)** Hypothesized pathway for 1-nonene biosynthesis in maize. **(B)** RT-qPCR analysis for the potential biosynthetic genes of the 1-nonene [*probable phospholipid-transporting ATPase* 8 (LOC103643332)]. HN, aphid-infested maize in high nitrogen; LN, aphid-infested maize in low nitrogen.

**Figure 9:**
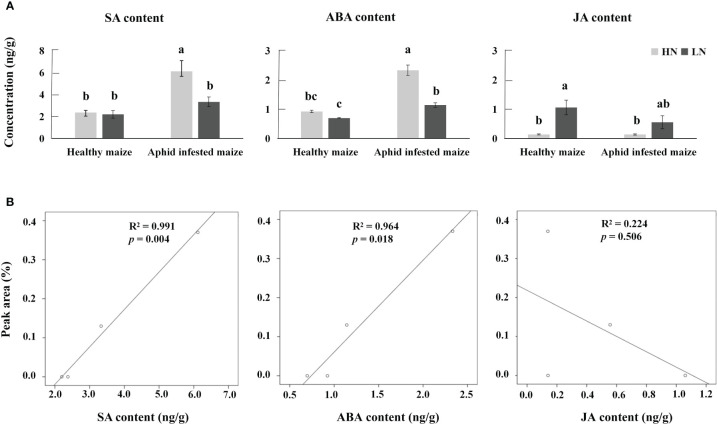
**(A)** Average concentrations of jasmonic acid (JA), salicylic acid (SA), and abscisic acid (ABA) in aphid-infested and healthy maize under high-nitrogen (HN) and low-nitrogen (LN) conditions. Different letters above the bars indicate significant differences (the phytohormone multiple comparisons was used Tukey’s test). **(B)** Correlation analysis between SA, ABA, JA, and 1-nonene.

The biosynthetic pathways of 1-nonene are not yet known; however, some biosynthetic enzymes (OleT_JE_/P450 decarboxylase, UndA/nonheme iron decarboxylase, and UndB/membrane-bound desaturase-like decarboxylase) from bacteria can convert free fatty acids to 1-alkenes, providing insights into the biosynthetic pathways of 1-nonene (Liu and Li, 2020). To fill this knowledge gap, we proposed a synthetic pathway for 1-nonene in maize B73 based on the biosynthesis of the fatty acid-derived hydrocarbons described above (**Figure 8A**). Based on homology analysis by the online program MaizeGDB BLAST (https://staging.maizegdb.org/) using the maize B73 genome, a probable *phospholipid-transporting ATPase 8* (GenBank No: LOC103643332), showing relatively high homology (41.95%) with OleT_JE_, was selected as the candidate gene for 1-nonene biosynthesis in maize B73. Consistent with the amount of 1-nonene emitted, the expression level of the *phospholipid-transporting ATPase 8* in infested maize under high-nitrogen conditions was higher than that in infested maize under low-nitrogen conditions (*t* test: *df*=4, *t*=6.602, *p* = 0.0027) (**Figure 8B**). To investigate whether stress-related phytohormones were activated, the concentrations of JA, SA and ABA in maize leaves were determined. The accumulation of SA was significantly increased following aphid infestation under high-nitrogen conditions (Tukey’s test, *p*< 0.001) (**Figure 9A**), but there is no significant difference for the SA concentration between the healthy maize under HN conditions and LN conditions (Tukey’s test, *p* = 0.996), and there is no significant difference between health maize and aphid-infested maize under LN conditions (Tukey’s test, *p* = 0.646). Similarly, the concentration of ABA in the aphid-infested maize under HN conditions were obviously increased (Tukey’s test, *p*< 0.001), and there is significant difference between health maize and aphid-infested maize under LN conditions (Tukey’s test, *p* = 0.016), but there is no significant difference between HN conditions and LN conditions in the healthy maize (Tukey’s test, *p* = 0.379). However, the concentration of JA in health maize under LN conditions was higher than health maize under HN conditions (Tukey’s test, *p* = 0.003), and also higher than aphid-infested maize under HN conditions (Tukey’s test, *p* = 0.003), but there is no significant difference between aphid-infested maize under LN conditions and aphid-infested maize under HN conditions (Tukey’s test, *p* = 0.319). SA (R^2 ^= 0.991, *F*= 232.745, *p* = 0.004) and ABA (R^2 ^= 0.964, *F*= 53.977, *p* = 0.018) showed significantly positive correlation with volatilization of 1-nonene, but, there is no obvious correlation with JA (R^2 ^= 0.244, *F*= 0.646, *p* = 0.280) (**Figure 9B**).

## Discussion

VOCs serve as attractants for predators play an important role in tritrophic interactions (Hiltpold et al., 2011; Aartsma et al., 2017; Turlings and Erb, 2018). The present study reveals that 1-nonene affect tritrophic interactions indirectly via nitrogen changes in the aphid-infested maize. This finding adds a new dimension to the role of HIPVs as mediators of tritrophic interactions.

Herbivore-induced plant volatiles (HIPVs) were usually used to locate the food source for predatory insects in plant-herbivores-natural enemies tritrophic interactions systems. For example, the females of parasitoid (*Cotesia marginiventris*) were attractive by the volatiles from corn seedlings which beet armyworm larvae were feeding in the flight tunnel bioassays. Besides, some researchers found that nitrogen content can mediated plant- herbivores-natural enemies tritrophic interactions systems. Some researchers found that natural enemies preferred plants that growing on a higher nitrogen condition by detected plant volatile cues (Loader and Damman, 1991; Zhu et al., 2020a). The amount of volatile secondary substances released by the host plant was much greater than the amount of secondary substances released by the pest itself, which provided natural enemies with reliable information about the possible presence of the host plant pest (Turlings et al., 1991). Therefore, we have collected and identified leaf volatiles from host plants in HN/LN conditions. The result showed that the composition of volatiles was significantly altered under different nitrogen levels.

Nitrogen levels affect the levels of plant hormones and the expression of numerous genes involved in metabolite synthesis, which subsequently results in changes in plant volatiles that might be the key signals used by natural enemies to locate pests (Chen et al., 2008; Kutyniok and Müller, 2013). For example, rice plants treated with different nitrogen levels showed changes in the composition of volatiles, thereby regulating the foraging and searching behavior of *Cyrtorhinus lividipennis* (Lou and Cheng, 2003; Zhu et al., 2020b), which consistent with our research. Many studies have shown that higher nitrogen level could enhance capability of parasitism of natural enemies and plant defense (Aqueel et al., 2014; Chesnais et al., 2016; Zhu et al., 2020a). For example, Sun et al. found that rice resistant response to brown rice planthopper (BPH) was obviously affected under different nitrogen condition by regulation of callose content and volatile emission, resulting in BPH preferred rice plants under higher nitrogen levels (Sun et al., 2020).

Our results found that *H. axyridis* was more attracted to volatiles emitted by aphid-infested maize under high-nitrogen conditions than under low-nitrogen conditions. Our study also found differences in the levels of the seven compounds between high- and low-nitrogen conditions. Seven volatile compounds showed significant differences among HN/LN conditions, such as benzaldehyde, 1-nonene, acetic acid, and indole, the levels of which were higher under HN than under LN; (-)-aristolene, azulene, and caryophyllene were produced in higher amounts under LN than under HN. This demonstrated that the same VOCs in different quantities could function as preferred host plant cues, depending upon the context in which they were perceived (Pingyan, 2009). So we quantified the differential volatiles and simulated the volatilities under natural conditions. 1-nonene may affect tritrophic interactions systems. When *H. axyridis* are exposed to mixed volatiles, supplementation with 1-nonene to HN levels can still change *H. axyridis* behavior. Previously reported that herbivory-induced indole increases the recruitment of the solitary endoparasitoid *Microplitis rufiventris* to maize plants that are induced by *Spodoptera littoralis* caterpillars. Indole exposure rendered the body odors of the caterpillars significantly less attractive, an effect that persisted even in the presence of more attractive host plants, which consistent with our research (Ye et al., 2018).

1-Nonene was found in pear, peach trees (Najar-Rodriguez et al., 2013) and *Senecio madagascariensis* (Kashiwagi et al., 2022), it was an attractant for males *Aedes aegypti* (L.) (Diptera: Culicidae). It is found that 1-nonene was the active compound in maize HIPVs that attracted *H. axyridis* that responded to nitrogen. The biosynthesis pathways of 1-nonene are not yet known, however, some biosynthetic enzymes (OleTJE/P450 Decarboxylase, UndA/non-heme iron decarboxylase, UndB/membrane-bound desaturase-like decarboxylase) able to convert free fatty acids to 1-alkenes from Bacteria provided insights into biosynthesis pathways of 1-nonene (Liu and Li, 2020). Based on the results of previous studies (Rude et al., 2011; Zhe et al., 2014; Zhe et al., 2015; Liu and Li, 2020), we predicted the synthesis pathway of 1-nonene. The *in vivo* bioconversion studies along with cell-free *in vitro* olefin biosynthesis studies suggest that *Jeotgalicoccus* utilizes fatty acid intermediates as substrates for olefin biosynthesis and that fatty acid decarboxylation is a primary mechanism of terminal olefin production (Rude et al., 2011). The genes in Maize B73 with high homology to the biosynthesis genes of 1-alkenes above were supposed to possibly participate in biosynthesis of 1-nonene. Finally, the gene probable *phospholipid-transporting ATPase* 8 in maize B73 showing high homolgy with OleTJE/P450 Decarboxylase was selected as potential biosynthesis genes of 1-nonene. Overall, the signaling pathway for the synthesis of 1-nonene is a complex process that includes the interaction of multiple links. By studying these links and their interactions in depth, the mechanisms of 1-nonene synthesis can be better understood and provide strong support for maize tritrophic interactions. The manipulation of 1-nonene biosynthesis allows for precise assessments of HIPV effects in the field and demonstrates that 1-nonene may be beneficial for maize in the context of multitrophic interactions. Interdependence of 1-nonene biosynthesis/action and fatty acyl-CoA reductaseas/oxidative decarbonylase expression is an interesting prospect for future research. How to reconcile the tritrophic interactions with the nitrogen fertilizer and how to make better use of plant volatiles to regulate the behavior of *H. axyridis* to achieve more desirable biological control effects still needs to be studied.

In addition to VOCs and hormones, the nitrogen levels have a significant effect on the growth and development of maize. The root length, root weight, shoot length, shoot weight and crop production increased under high-nitrogen condition (Chen et al., 2008; Schluter et al., 2012; Kutyniok and Müller, 2013; Chesnais et al., 2016), which was in consistant with our previous study as well (Zhao et al., 2022). Similarly, nitrogen levels affect natural enemy insects, it has been reported that improvement in N inputs enhances parasitism, the growth and development of parasites (Kaneshiro et al., 1996; Aqueel et al., 2014). It was also shown high N level positively promotes performances of phytophagous insects in terms of population growth rate and weight increase (Aqueel and Leather, 2011). In this study, aphid-infested maize under high N condition showed stronger attractive to the *H. axyridis* compared to the maize under low N condition. Our previous study showed *Rhopalosiphum padii* preferred the maize grown under the high-nitrogen condition (Zhao et al., 2022). Base on results from the previous and our studies, we proposed that the both of pests and their enemies tend to targets with abundant nutrition, such as the maize under high N condition and more aphids attracted by the maize under high N condition, even though predation risk is high for the aphids (*R. padi*) attracted by the maize under high N condition.

**Figure 10:**
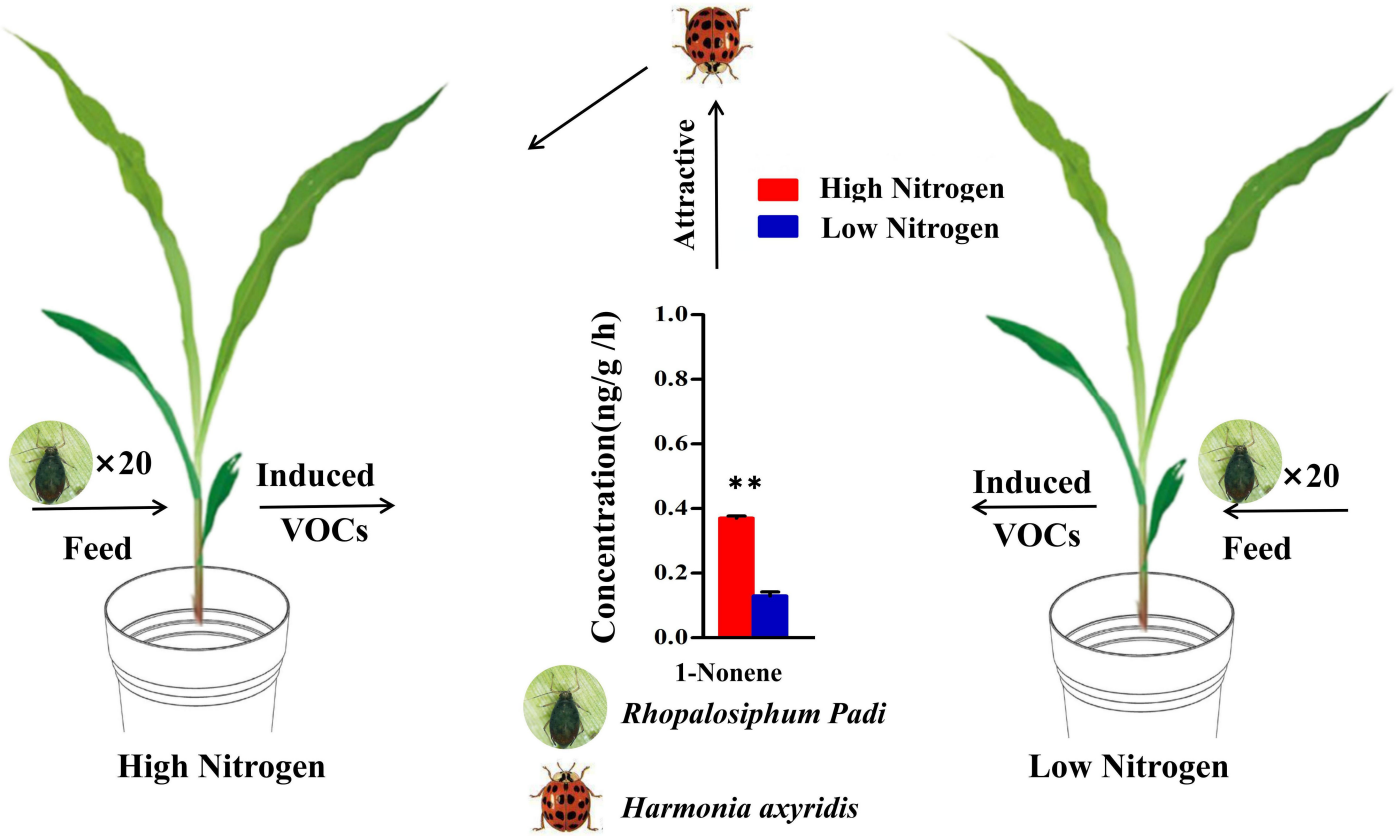
Model of the tendency of *H. axyridis* to visit maize under high/low-nitrogen conditions. (Student’s *t*-test: **p< 0.01).

Overall, our study further revealed the key role of 1-nonene when aphids fed on maize in the response of maize-aphid-ladybird tritrophic interactions to nitrogen (**Figure 10**). These findings may have practical importance for the development of habitat manipulation strategies, given that HIPVs induced by aphid feeding are important signals. Meanwhile, this study also provides a novel perspective and strategy for maize pest resistance research and integrated pest management. However, there is also need to comprehend the molecular and biochemical mechanisms in the production and recognition of the key VOCs, which probably offer new insights for molecular breeding and environment-friendly pest control.

## Data availability statement

The original contributions presented in the study are included in the article/**Supplementary Material**. Further inquiries can be directed to the corresponding authors.

## Author contributions

S-WZ: Data curation, Methodology, Validation, Writing – original draft, Writing – review & editing. J-HX: Funding acquisition, Project administration, Writing – review & editing. YP: Methodology, Software, Writing – original draft. ZW: Software, Writing – original draft. XW: Writing – original draft. SW: Formal Analysis, Project administration, Validation, Writing – review & editing.
