# Supplementary material for: 1-nonene plays an important role in the response of maize-aphid-ladybird tritrophic interactions to nitrogen
Source: Front Plant Sci. 2024 Jan 8;14:1296915. doi: 10.3389/fpls.2023.1296915 (PMC10800950; doi:10.3389/fpls.2023.1296915)
Supplement: Supplementary file 1 [file DataSheet_1.docx]

**Supporting information for:**

**1-Nonene plays an important role in the response of maize-aphid-ladybird tritrophic interactions to nitrogen**

**Shi-Wen Zhao^1^, Yu Pan^1^, Zhun Wang^2^, Xiao Wang^1^, Shang Wang^1^*, Jing-Hui Xi^1^*,**

1College of Plant Science, Jilin University, Changchun, China

2Changchun Customs Technology Center, Changchun, China

* Corresponding author.

1. mail address: shangwang@jlu.edu.cn

[jhxi1965@jlu.edu.cn](mailto:jhxi1965@jlu.edu.cn)

Title page

Supporting Information:

Table S1. Primers of RT-qPCR used in this study.

Figure S1. GC-FID trace of the headspace volatiles of the maize variety B73 infested by aphids in high nitrogen condition (a) and gas chromatography analysis of authentic 1-nonene (b).

Figure S2. (a) Mass spectrum of 1-nonene from the NIST (National Institute of Standards and Technology, Washington D.C., U.S.) spectral database. (b) Mass spectrum of the authentic 1-nonene standard. (c) Mass spectrum of the compound, identified as 1-nonene, from the maize variety B73 infested by aphids.

Figure S3. Mass spectrum of 4-nonene (a), 3-nonene (b), 2-nonene (c), cis-2-nonene (d) from the NIST (National Institute of Standards and Technology, Washington D.C., U.S.) spectral database.

**Supplementary Table 1. Primers of RT-qPCR used in this study**

| Gene Name | Forward primer | Reverse primer |
| --- | --- | --- |
| LOC103643332 | GTGTTATCGTCGGATTGCAGC | CAGTACAGTGCCGAGAGGAC |


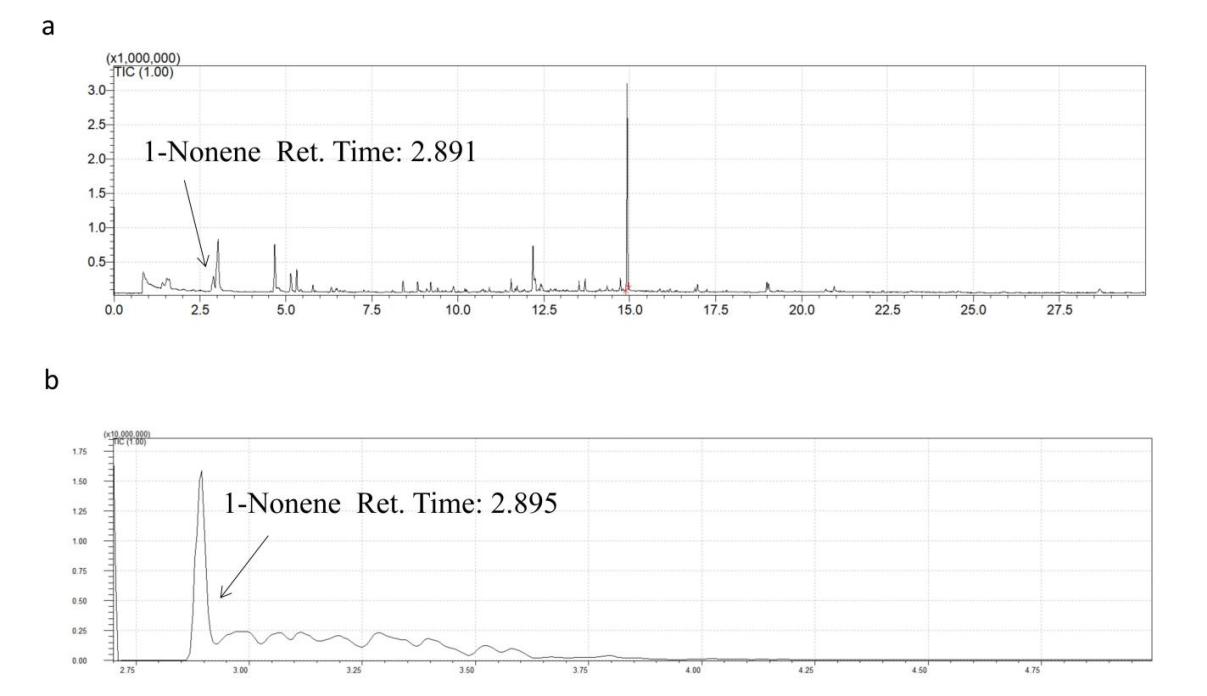


Figure S1. GC-FID trace of the headspace volatiles of the maize variety B73 infested by aphids in high nitrogen condition (a) and gas chromatography analysis of authentic 1-nonene (b).


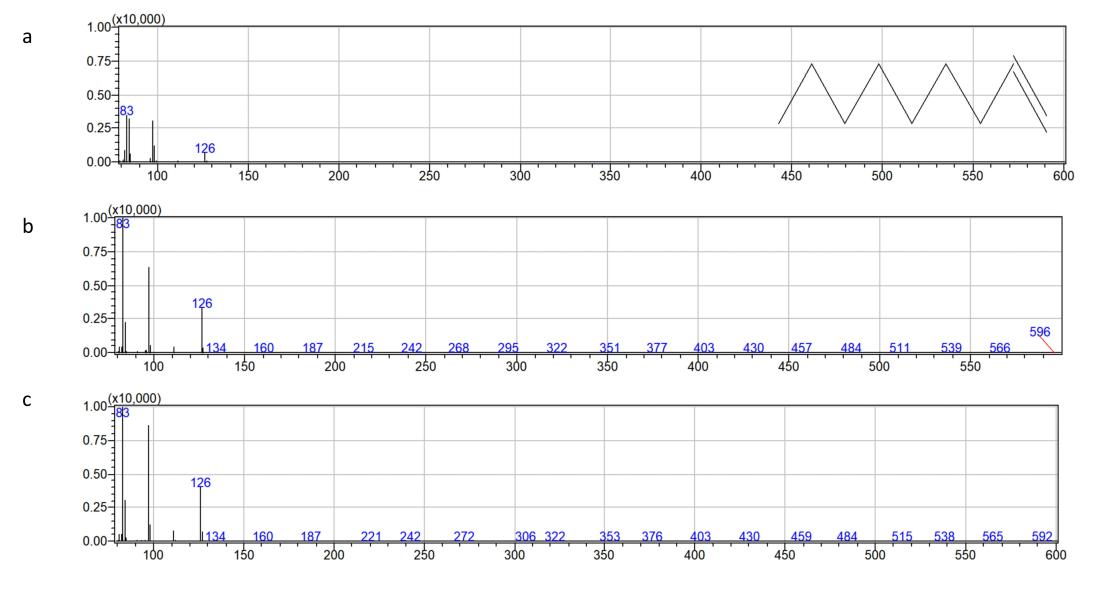


Figure S2. (a) Mass spectrum of 1-nonene from the NIST (National Institute of Standards and Technology, Washington D.C., U.S.) spectral database. (b) Mass spectrum of the authentic 1-nonene standard. (c) Mass spectrum of the compound, identified as 1-nonene, from the maize variety B73 infested by aphids.


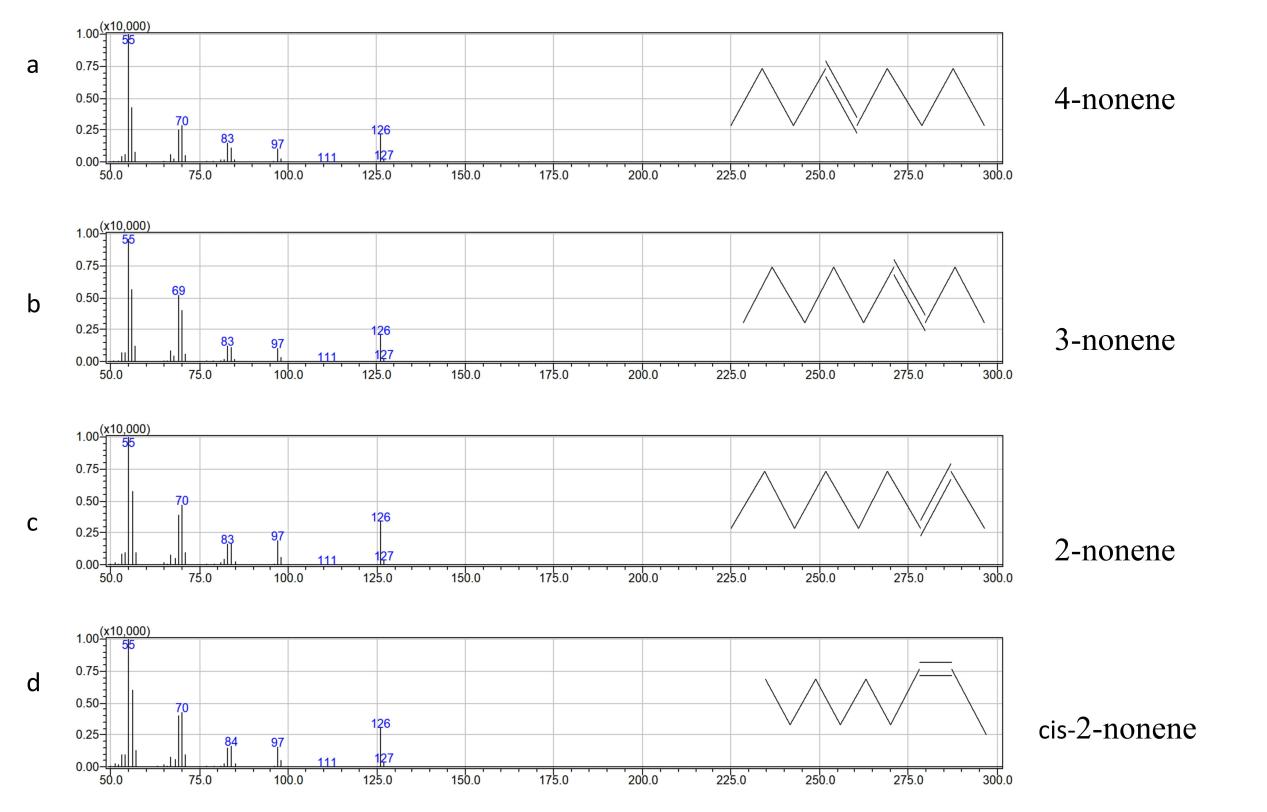


Figure S3. Mass spectrum of 4-nonene (a), 3-nonene (b), 2-nonene (c), cis-2-nonene (d) from the NIST (National Institute of Standards and Technology, Washington D.C., U.S.) spectral database.
